# Molecular characterization and gene expression analysis of the pro-inflammatory cytokines IL-1β and IL-8 in the South American fish *Piaractus mesopotamicus* challenged with *Aeromonas dhakensis*

**DOI:** 10.1590/1678-4685-GMB-2020-0006

**Published:** 2020-11-06

**Authors:** Mateus Maldonado Carriero, Flavio Henrique-Silva, Caroline Munhoz Meira, Igor Mateus Queiroz Gato, Alexandre Rodrigues Caetano, Francisco Pereira Lobo, Anderson Luis Alves, Eduardo Sousa Varela, Antonio Augusto Mendes Maia

**Affiliations:** ^1^ Universidade de São Paulo, Faculdade de Zootecnia e Engenharia de Alimentos, Departamento de Medicina Veterinária, Pirassununga, SP, Brazil.; ^2^ Universidade Federal de São Carlos, Departamento de Genética e Evolução, São Carlos, SP, Brazil.; ^3^ Embrapa Recursos Genéticos e Biotecnologia, Empresa Brasileira de Pesquisa Agropecuária, Brasília, DF, Brazil.; ^4^ Embrapa Informática na Agricultura, Empresa Brasileira de Pesquisa Agropecuária, Campinas, SP, Brazil.; ^5^ Embrapa Pesca e Aquicultura, Empresa Brasileira de Pesquisa Agropecuária, Palmas, TO, Brazil.

**Keywords:** Bacterial infection, fish immunology, gene expression, interleukin, South American fish

## Abstract

In the present study, the complete characterization of cDNA and genomic sequences of IL-1β and IL-8, as well as the expression profile of these genes in the South American fish pacu (*Piaractus mesopotamicus*) is provided. The full-length pmIL-1β cDNA was composed of 1208 nucleotides that would produce a precursor peptide with 273 amino acid residues. A putative caspase-1 cleavage site, similar to what is found in mammalian IL-1β, was identified producing a mature peptide with a theoretical molecular weight of 17.21 kDa. The pmIL-8 cDNA sequence consisted of 1019 nucleotides which encoded a 95-amino acid protein with a theoretical molecular weight of 10.43 kDa that showed all typical CXC chemokine features, including a 20-residue signal peptide and four conserved cysteine residues. Constitutive mRNA expression was detected for both genes in the liver, head kidney, gill, intestine, skin and spleen. After a bacterial challenge, up-regulation was detected for both pmIL-1β and pmIL-8 in the spleen and head kidney at 12 h post-infection. At 24 h post-infection there was a decrease in the expression of both genes, with pmIL-8 showing a significant down-regulation in the liver and head kidney when compared to the control groups.

## Introduction

Aquaculture is a constantly developing economic activity in Brazil and the characiform *Piaractus mesopotamicus* (Holmberg 1887), commonly known as “pacu”, is the second most produced native species with increasing importance in global aquaculture due to its desired characteristics such as easy reproduction, ecological value and marketability (Witmer and Fuller, 2011; MPA, 2013; Lin *et al.*, 2015).

However, the increase of its production in fish farms, frequently in intensive systems, facilitates the occurrence of disease outbreaks, leading to substantial economic losses. Among the infectious diseases commonly observed in farmed pacus, some of the most important are the ones caused by bacteria of the *Aeromonas* genus, which have been associated with septicemia-induced mortality outbreaks in this species in Brazil (Carriero *et al.*, 2016; Claudiano *et al.*, 2019; Marinho-Neto *et al.*, 2019). Furthermore, the lack of knowledge regarding immune responses in *P. mesopotamicus* prevents the development of appropriate treatments and prophylactic strategies to decrease the frequency in which these outbreaks occur (Farias *et al.*, 2018).

Pro-inflammatory cytokines are a class of small proteins involved in the communication between cells of the immune system that are generally characterized as key initiators of inflammatory processes after the detection and recognition of infections (Secombes *et al.*, 2011; Pleic *et al.*, 2014). Two of the most studied pro-inflammatory cytokines are the interleukin-1β (IL-1β) and interleukin-8 (IL-8) due to their critical importance in the initiation and maintenance of inflammations (Dinarello, 1997; Kendrick *et al.*, 2014).

Several studies have shown the importance of IL-1β and IL-8 in the regulation of inflammatory responses during bacterial and parasitic infections, and the analysis of their expression is recognized as good indicators of the occurrence of inflammatory processes (Morrison, 2012; Herath *et al.*, 2016; Wang *et al.*, 2017; Wang *et al.*, 2019). Recently, these cytokines have also been recognized as potential molecular adjuvants in fish vaccination, with promising results already obtained against viral and bacterial pathogens (Peddie *et al.*, 2003; Jimenez *et al.*, 2006; Sanchez, 2007; Wang *et al.*, 2016; Wang *et al.*, 2019), further increasing the importance of studies providing their structural and functional characterization.

In mammals, IL-1β is produced as an inactive precursor molecule (proIL-1β) that needs to be cleaved by the proteolytic enzyme caspase-1, also known as IL-1β converting enzyme (ICE) in a conserved aspartic acid (Asp) residue, usually between the positions 113 and 117, to produce the bioactive form, which is then secreted by the cells (Secombes *et al.*, 1999; Zou *et al.*, 1999). In fish species, the exact mechanism involved in the cleavage of proIL-1β into the mature biologically active form is still not fully elucidated. Although the caspase-1 cleavage site reported for humans is usually absent, for some species such as sea bass (*Dicentrarchus labrax*), a caspase-1-dependant cleavage mechanism has been described for the production of mature IL-1β peptide, however, in different aspartic acid residues (Reis *et al.*, 2012).[Bibr B46]

IL-8, also known as CXCL-8, is a small (8-12 kDa) chemokine produced by several immune related cell types such as lymphocytes, macrophages, peripheral blood mononuclear cells and epithelial cells (Goodman *et al.*, 1991; Nakamura *et al.*, 1991; Mukaida *et al.*, 1998). It is involved in the chemotactic attraction of immune cells during inflammatory reactions, mainly recruiting neutrophils and other important leukocytes to the infection or injury location (Li and Yao, 2013). In mammalian IL-8, a three-amino acid residue motif (Glu-Leu-Arg) is commonly found preceding the first cysteine (ELR^+^ CXC). The presence of this motif confers to proteins the ability to exert a chemoattractant effect on leukocytes, as well as to promote angiogenesis (Allen *et al.*, 2007; Harun *et al.*, 2008; Herath *et al.*, 2016). In teleost fish, however, this motif has not been identified in any species, with the exception being haddock (*Melanogrammus aeglefinus*), which has led to the speculation of whether fish IL-8 might have fewer functions than their mammalian counterparts, a hypothesis that is still not confirmed (Seppola *et al.*, 2008).

Most of the studies investigating the structural and functional characteristics of fish IL-1β and IL-8 have been performed mainly with representatives of the Salmoniformes, Cypriniformes, Siluriformes and Perciformes orders, with no studies providing a detailed analysis of the structure and expression analysis under pathogenic infections of IL-1β and IL-8 of fish from the Characiformes order being found in the literature.

Therefore, the present study aims to provide the characterization of the complete cDNA sequences, as well as the genomic regions, of the cytokines IL-1β and IL-8 from the South American fish pacu. Complete sequence analysis including a phylogenetic comparison of the obtained sequences with the sequences from other vertebrates, domain architecture and secondary and tertiary structures of the deduced proteins are provided. Moreover, the tissue distribution and expression profile of these cytokines in the liver, spleen and head kidney of bacteria-infected fish were investigated.

## Materials and Methods

### Ethics statement

All animal handling was performed following the Brazilian animal welfare guidelines (Federal Law n° 11.794 dated October 8^th^, 2008) and the present study was approved by the ethics committee for animal welfare of the Faculty of Animal Science and Food Engineering of the University de São Paulo (FZEA/USP) under the protocol number 14.1.391.74.9.

### Experimental fish

Pacus (weighing 33.8 ± 4.8 g) used in the present experiment were purchased from a local fish farm and maintained in 40 L glass tanks with filtered dechlorinated tap water under constant aeration with controlled temperature at 27 ± 2 °C and with a controlled photoperiod of 12 h:12 h of light/darkness. Fish were fed twice a day with a commercial dry pelleted feed until satiation. A third of the water of each tank was replaced every two days to prevent accumulation of organic matter. Fish were maintained under these conditions for three weeks prior the experiments for acclimation.

### Pacu IL-1β and IL-8 cDNA and genomic DNA cloning

For the initial characterization of the genes IL-1β and IL-8, total RNA was isolated from approximately 30 mg of head kidney, spleen and liver using TRIzol^®^ Reagent (Thermo Fisher Scientific) following the manufacturer's protocol, quantified in a NanoDrop 2000 (Thermo Scientific) spectrophotometer at 260 nm and analyzed by agarose gel electrophoresis to assess the integrity of the samples. Prior to reverse transcription reactions, in order to eliminate possible genomic DNA contamination, samples were treated with 1 unit of RQ1 RNase-free DNase (Promega) per μg of RNA. DNase treated RNAs were then subjected to cDNA synthesis using the High Capacity Reverse Transcription Kit (Applied Biosystems) following manufacturer's instructions.

For the initial amplification of *P. mesopotamicus* IL-1β and IL-8 (pmIL-1β and pmIL-8) fragments, primers were designed based on conserved regions of the predicted sequences of the red-bellied piranha (*Pygocentrus nattereri*, GenBank accession numbers XM_017693723 for IL-1β and XM_017685589 for IL-8) and tambaqui (*Colossoma macropomum*) (unpublished data), other South American characiforms from the Serrasalmidae family (Table 1). Amplified fragments were purified, ligated to the pGEM^®^-T Easy Vector (Promega) and transformed into DH5α *Escherichia coli* competent cells, which were then inoculated on LB-agar plates supplemented with 100 μg/mL ampicillin, 0.5 mM IPTG and 50 mg/mL X-Gal. Ligated plasmids were recovered using the PureYield^TM^ Plasmid Miniprep System (Promega) and sequenced using the T7 – SP6 primers as well as specific internal primers for each gene (Table 1).

**Table 1:** Primers used for *P. mesopotamicus* IL-1β and IL-8 cloning and expression analysis.

Primer	Target gene	Sequence (5’-3’)	Product size	Use
IL-1βF1	IL-1β	ACTGGACTGCTCTGATCCTTTG	639 bp	Initial amplification
IL-1βR1		CAGCCTGGGTACTTCACTGATT		
IL-1βF2		ACCGTGTGTGACAGCTTAAAGA	94 bp	qPCR and RACE
IL-1βR2		TTTGGCTACTGTTTCCACCCTT		
IL-1βF3		GAGCTGAAAGACTACAGCTAAACT	2021 bp	gDNA amplification
IL-1βR3		GTCTTGGACGTTACAACACCAG		
IL-8F1	IL-8	GTGGGGGTGTGTTTATTTTTGG	288 bp	Initial amplification
IL-8R1		ATCTTGTGTCTGACCTTAGGGTG		
IL-8F2		AGACGGATCGGCAAACTGATAGA	90 bp	qPCR and RACE
IL-8R2		GTTCTTTAGCGTCGCTATGATCT		
IL-8F3		AGCTGAAACTCCAGACACAGTC	2045 bp	gDNA amplification
IL-8R3		TGTCCAAAGTTACTCATGCCTCT		
BactF	β-actin	TCACAGAGGCTCCCCTGAAC	64 bp	qPCR
BactR		CTCAAACATGATCTGGGTCATCT		
T7	Vector	TAATACGACTCACTATAGGG	-	Plasmid sequencing
SP6		TATTTAGGTGACACTATAG		

The complete cDNA sequences of pmIL-1β and pmIL-8 were amplified using the 3’ and 5’ RACE System for Rapid Amplification of cDNA Ends Kits (Invitrogen), cloned and sequenced as described above. For characterization of the complete genomic sequences, gDNA was isolated from pacu tail fin clips using the Dneasy^®^ Blood & Tissue Kit (Qiagen) following the manufacturer's instructions and amplified using primers designed based on the full-length cDNA of pmIL-1β and pmIL-8 (Table 1).

### *P. mesopotamicus* IL-1β and IL-8 sequence analysis

The obtained full-length cDNA sequences were translated to obtain the putative amino acid sequences of pmIL-1β and pmIL-8 using the online Sequence Manipulation Suite (Stothard, 2000) (https://www.bioinformatics.org/sms/index.html). The exon/intron structure of the genomic sequences was determined by comparing the obtained cDNA and genomic sequences using the Splign software (Kapustin *et al.*, 2008).

Theoretical isoelectric points (pI) and molecular weights (Mw) of the deduced amino acid sequences were estimated using the Compute pI/Mw tool and characteristic signature domains and structures were predicted using the Prosite server on the ExPASy Bioinformatics Resource Portal (http://web.expasy.org/compute_pi/). Disulfide bonds were predicted using the DiANNA web server for cysteine classification and disulfide connectivity prediction (Ferre and Clote, 2006).

The tertiary structure of pmIL-1β and pmIL-8 was predicted using the online SWISS model interface (Arnold *et al.*, 2006) with the 2.10 Å resolution human IL-1β crystal structure (SMTL ID 5i1b.1) and the human 2.00 Å resolution IL-8 crystal structure (SMTL ID 3il8.1), respectively. Three-dimensional (3D) images were visualized and edited using UCSF Chimera molecular graphics software version 1.13.1 (Pettersen *et al.*, 2004).

Multiple sequence alignments were produced using Clustal W (Thompson *et al.*, 1994) to analyze the relationship between pmIL-1β and pmIL-8 amino acid sequences and other vertebrate species. Neighbor Joining phylogenetic analysis was performed based on the Poisson correction model and bootstrapped 1000 times using MEGA 7 software (Kumar *et al.*, 2016).

### Bacterial challenge

For the experimental infections, a pathogenic strain of *Aeromonas dhakensis*, which was previously isolated from diseased pacu fish during an outbreak in Brazil (Carriero *et al.*, 2016), was cultured for 24 h at 30 °C in Tryptic Soy Broth (TSB) (BD Difco^TM^) containing 10 mg/mL of ampicillin with constant shaking until an optical density of 0.4 at 600 nm was obtained. This culture was then diluted to the concentration of 4.4 × 10^4^ CFU in 0.1 mL of sterile 0.9% NaCl saline solution. This inoculum, which represented the lethal dose for 20% of the infected fish for this bacterial strain as previously described, was administered to each fish by intraperitoneal injection (Carriero *et al.*, 2016, 2018). Fifty pacus were inoculated with 0.1 mL of the bacterial inoculum and randomly divided into five 40 L glass tanks to a final density of ten fish per tank. For the control group, 50 pacus were injected with 0.1 mL of sterile 0.9% NaCl saline solution.

During the experimental period, seven fish from the infected group died after the bacterial inoculation. These fish were immediately removed from the tanks and processed for re-isolation of the bacteria by inoculating macerated fragments of organs on Tryptic Soy Agar (TSA) (BD Difco^TM^) with no antibiotics, followed by PCR amplification with *Aeromonas* specific primers to confirm the infection (Yanez *et al.*, 2003). Also, several specimens that survived the infection and were used in the gene expression experiment showed clinical signs associated with *Aeromonas*-induced acute hemorrhagic septicemia, which included hemorrhagic foci in the eyes, gills, fins and anal region, abdominal swelling, erratic swimming and darkening of the skin (Carriero *et al.*, 2016). No fish from the control group showed any clinical signs or clear behavioral alteration.

After the experiments, five fish from the control group were tested using the same isolation protocol, with no bacterial DNA being detected (data not shown).

### *P. mesopotamicus* IL-1β and IL-8 tissue distribution and expression analysis following infection with A. dhakensis

To evaluate transcript levels of the cytokines IL-1β and IL-8 under physiological conditions, mRNA was quantified in the liver, spleen, head kidney, gills, intestine and skin of five healthy uninfected pacus. At the time of sampling, these fish were euthanized by lidocaine overdose, visually analyzed for the presence of any signs of infections, such as excess of mucus and changes in the color of skin and gills and hemorrhagic foci in the internal organs, as well as for the occurrence of any parasitic infection, and fragments of each organ were aseptically collected.

To assess the role of these interleukins during the infection with the bacterium *A. dhakensis* in pacu, five fish from the infected and control groups were euthanized and fragments of liver, spleen and head kidney were aseptically collected at 12, 24 and 48 h post-infection. All samples were immediately frozen in liquid nitrogen and subjected to RNA extraction and cDNA synthesis as described above.

The determination of pmIL-1β and pmIL-8 mRNA levels was performed by quantitative real-time PCR (qPCR) using specific primers designed based on the obtained full-length cDNA sequences (Table 1). Prior to expression analyses, each primer pair was tested for amplification efficiency by creating a standard curve with serial dilutions of the cDNA of a representative sample using the formula E = (10^−1/slope^-1) × 100. This efficiency analysis was performed three times for each gene and the average efficiencies were 101.7% for IL-1β and 94.5% for IL-8. The same efficiency analysis was performed for the β-actin gene, which was used as endogenous reference with previously described primers (de Paula *et al.*, 2014).

Quantitative PCR (qPCR) reactions were performed in duplicates with a final volume of 10 μL consisting of 5 μL of GoTaq^®^ qPCR Master Mix, 300 nM of each primer, 10 ng of cDNA and MiliQ ultrapure water using a Quantstudio^TM^ 6 Flex Real-Time PCR System (Applied Biosystems). A no-template control (NTC) reaction was performed for each gene on each run and, immediately after every amplification, a melt curve analysis was performed to verify the occurrence of unspecific amplification or primer dimers.

The relative expression levels were obtained by subtracting the mean Ct values of the target genes from the mean β-actin values (ΔCt) which were applied to the 2^−DCt^ algorithm (Schmittgen and Livak, 2008). For the basal expression analysis, data are presented as relative fold-change to the liver for IL-1β and to the intestine for IL-8, which were the organs with the lowest expression levels. For expression analysis of the bacteria-infected fish, data are presented as relative fold-change to the control samples at the same time point.

### Statistical analysis

Relative gene expression data were analyzed for variance (ANOVA) and the means were compared using the Student's *t-*test to assess the differences of gene expression between the infected and control groups at the same time point. The pmIL-1β and pmIL-8 distribution analysis among different tissues was performed using Tukey's test. The equality of variance was tested by Levene's test and the normality was assessed using the Kolgomorov-Smirnov test. Differences were considered statistically significant when *p* ≤ 0.05.

## Results

### Characterization and sequence analysis of *P. mesopotamicus* IL-1β and IL-8

**Figure 1:**
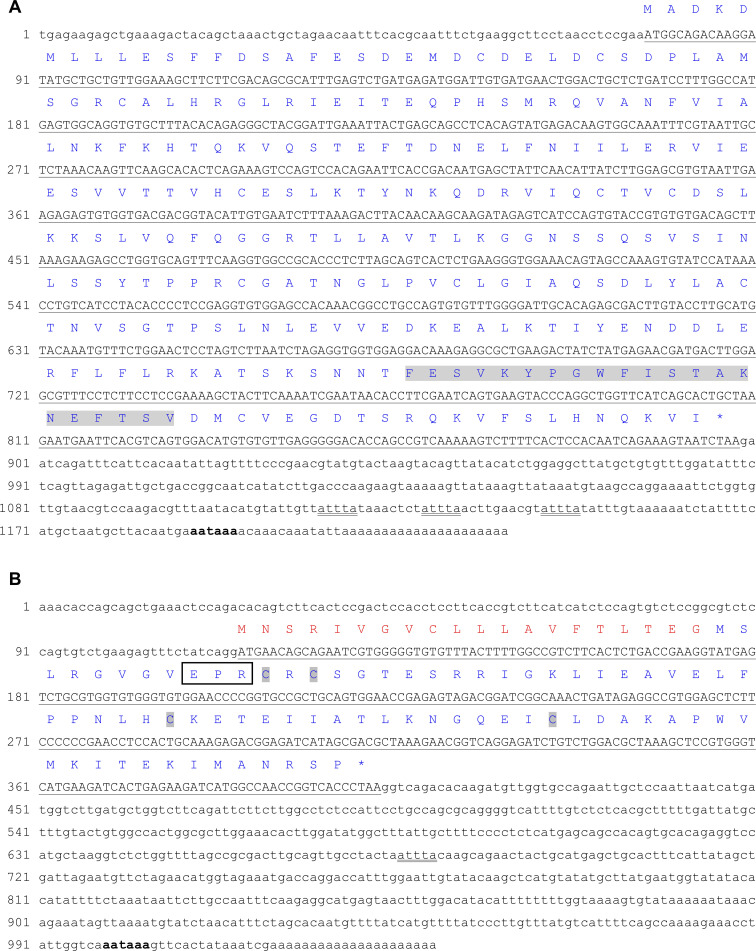
Full-length cDNA sequence of pmIL-1β (A) and pmIL-8 (B) with deduced amino acids. The ORFs are underlined and shown in uppercase letters and the 5’ and 3’ UTRs are shown in lowercase letters. (A) The IL-1 family signature is highlighted in grey. Three instability motifs (ATTTA) are double underlined and the polyadenylation signal sequence (AATAAA) 19 nt upstream the poly A tail is in bold. (B) The putative signal peptide is shown in red. The four conserved cysteines are highlighted in grey. The EPR motif is indicated by a black box. A single instability motif (ATTTA) is double underlined and the polyadenylation signal sequence (AATAAA) 21 nt upstream the poly A tail is in bold.

The full-length cDNA sequence of pmIL-1β (GenBank accession number MT787039) was composed of 1208 nucleotides, subdivided in a 5’ untranslated region (UTR) of 76 nt, an open reading frame (ORF) of 822 nt, and a 3’ UTR of 310 nt containing a polyadenylation signal (AATAAA) 19 nt upstream the poly A tail and three instability motifs (ATTTA), which are typically observed in mRNAs from inflammatory cytokines, indicating that the expression of these proteins is highly regulated (Scapigliati *et al.*, 2001) (Figure 1A).

The putative sequence of pmIL-1β precursor protein was composed of 273 amino acid residues with a theoretical molecular weight of 30.54 kDa and an isoelectric point of 5.14. A putative caspase-1 cleavage site was identified between Asp^114^ and Arg^115^ producing a mature active peptide composed of 157 amino acid residues with a theoretical molecular weight of 17.21 kDa and an isoelectric point of 8.25.

**Figure 2:**
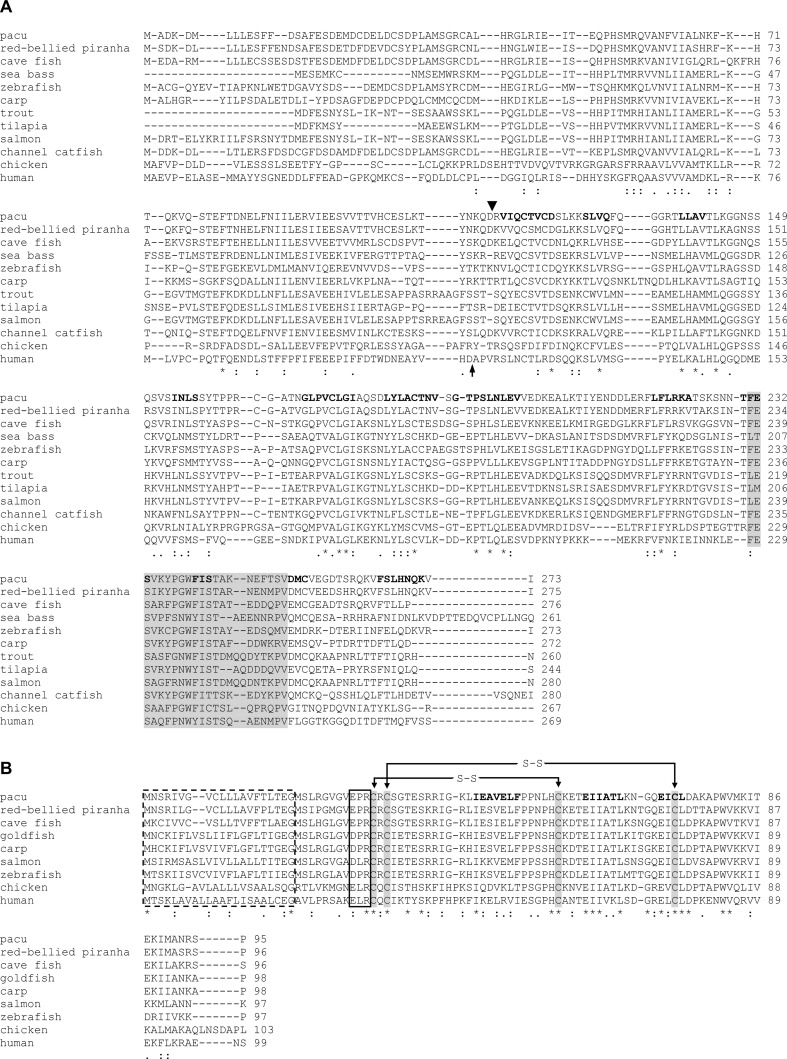
Multiple alignment of the predicted amino acid sequence of pmIL-1β and pmIL-8 with other known vertebrates IL-1β and IL-8 proteins. Identical (*) and similar (: or .) residues are indicated. (A) Putative caspase-1 cleavage sites are indicated with a downward arrow head for pacu IL-1β and with an upward arrow for human IL-1β. Conserved β-strand forming residues in the mature peptide are in bold. IL-1 family signature is indicated by grey shadow. (B) Signal peptide is indicated by a dotted box. The four conserved cysteines are highlighted by grey shadows. The EPR motif is indicated by a black box. Conserved β-strand forming residues in the mature peptide are in bold. Disulfide bonds are indicated by lines connecting sulfur atoms of cysteine residues (S-S).

The multiple sequence analysis showed that this putative caspase-1 cleavage aspartic acid observed in pmIL-1β is also present in the predicted sequences of the red-bellied piranha and cave fish, the other characiform species included in the analysis (Figure 2A). The only non-chaciform species with this aspartic acid in the same position was the channel catfish. However, the cleavage position of this species has not yet been subjected to experimental functional validation (Wang *et al.*, 2006).

The other regions of pmIL-1β were fairly conserved when compared to other vertebrates, especially in the predicted β-strands regions, with similarities ranging from 82.2% with red-bellied piranha to 25.9% with human IL-1β. The interleukin-1 signature motif was identified at positions 231 to 251 in the characterized by the sequence FeSVkyPgwFISTakneftsV (Figure 2A).

**Figure 3:**
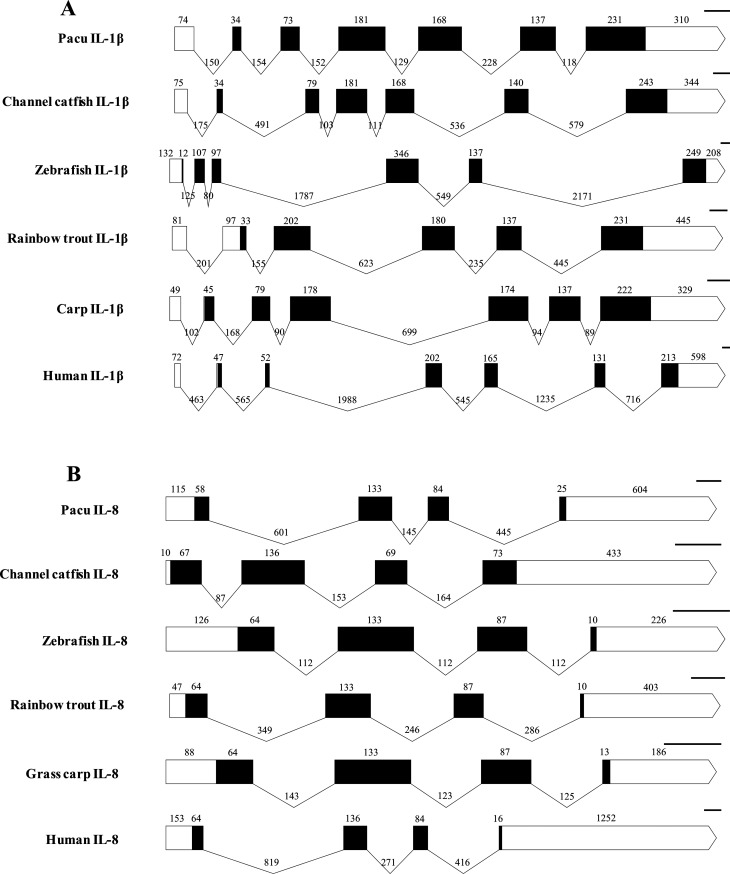
Schematic representations of exon-intron arrangement of pmIL-1β (A) and pmIL-8 (B). PmIL-1β was compared with the sequences of channel catfish (*Ictalurus punctatus* DQ157743), zebrafish (*Danio rerio* FM213388), raibow trout (*Oncorhyncus mykiss* AJ004821), carp (*Cyprinus carpio* AJ245635) and human (*Homo sapiens* M15840) and pacu IL-8 was compared with the sequences of channel catfish (*Ictalurus punctatus* AY145142), zebrafish (*Danio rerio* NC_007112), raibow trout (*Oncorhyncus mykiss* AY160987), grass carp (*Ctenopharyngodon idellus* JN663841) and human (*Homo sapiens* NG_029889). Exons are indicated by boxes and introns by v-shaped lines. White areas indicate untranslated regions (UTRs). The size of each exon and intron is given (in base pairs) above the boxes and bellow the lines, respectively. Scale bars = 100 base pairs.

The genomic region of pmIL-1β (GenBank accession number MT787041) showed 2139 nt in length and was structurally divided in seven exons and six introns, which is a similar structure to what is observed in the IL-1β from channel catfish, carp and human, but differed from zebrafish and rainbow trout, that showed only six exons and five introns. The seven exons of pacu IL-1β were 74, 34, 73, 181, 168, 137 and 541 nt in length, respectively. Similar to what was observed for almost all species analyzed, with the exception being zebrafish, the first exon of pacu IL-1β was entirely untranslated, with the start codon falling in the second exon (Figure 3A).

**Figure 4:**
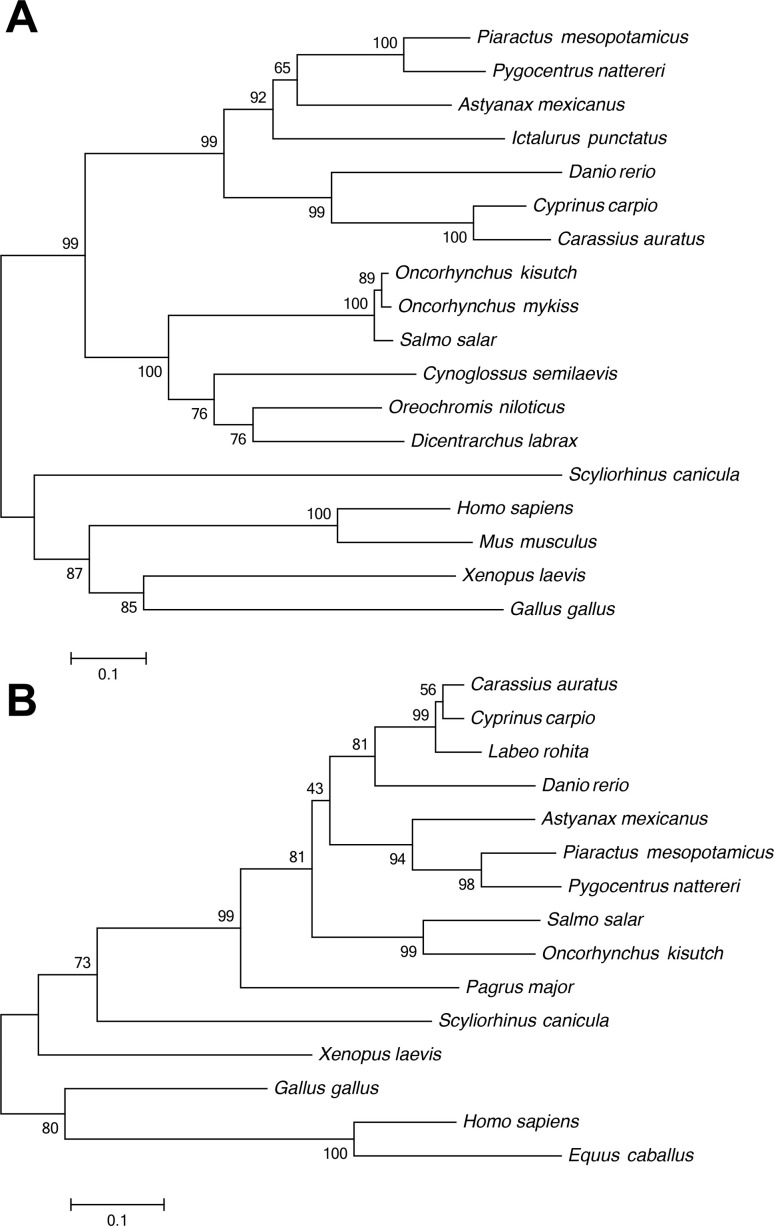
Neighbor Joining phylogenetic analysis comparing the complete amino acid sequences of pmIL-1β (A) with *Pygocentrus nattereri* XP_017549212, *Astyanax mexicanus* XP_022536473, *Ictalurus punctatus* NP_001187148, *Danio rerio* AAQ16563, *Cyprinus carpio* CAC19888, *Carassius auratus* CAD12102, *Oncorhynchus kisutch* XP_020331449, *Oncorhynchus mykiss* CAC83518, *Salmo salar* XP_014025954, *Cynoglossus semilaevis* ACU55137, *Oreochromis niloticus* XP_019221386, *Dicentrarchus labrax* CAC80553, *Scyliorhinus canicula* CAC80866, *Homo sapiens* NP_000567, *Mus musculus* EDL28238, *Xenopus laevis* AAI70521 and *Gallus gallus* NP_989855 and the sequence of sequence of pmIL-8 (B) with *Carassius auratus* AGG11027, *Cyprinus carpio* ABS70451, *Labeo rohita* ADJ53042, *Danio rerio* XP_009305130, *Astyanax mexicanus* XP_007229345, *Pygocentrus nattereri* XP_017541078, *Salmo salar* NP_001134182, *Oncorhynchus kisutch* XP_020360239, *Pagrus major* ADK35756, *Scyliorhinus canicula* CAD91126, *Xenopus laevis* AEB96252, *Gallus gallus* ADU60331, *Homo sapiens* AAH13615 and *Equus caballus* AAO37764. Numbers at branching points indicate bootstrap confidence levels for 1000 repetitions. Bar represents 0.1 amino acid substitutions per site.

The phylogenetic analysis revealed that the closest related species with pacu IL-1β was the red-bellied-piranha, followed by the cave fish, both members of the Characiformes order. The non-characiform species that was more closely related to pacu IL-1β was the channel catfish, a member of the Siluriformes order. Furthermore, there was a clear separation between the clade composed by teleost species clade from the one containing other vertebrates (Figure 4A).

These variations in exon/intron organization, however, produced little effect in the similarities observed between the resulting IL-1β peptides, as evidenced by the results in the phylogenetic analysis (Figure 4A), in which the phylogenetic proximity of the species was more determinant in the clustering than the exon/intron genomic structure. The closest related species with pmIL-1β was the red-bellied-piranha, followed by the cave fish, both members of the Characiformes order. Similarly, zebrafish and rainbow trout clustered together with other cypriniforms and salmoniforms, respectively, despite having different exon/intron organization than their closest counterparts.

The full-length cDNA sequence of pmIL-8 (GenBank accession number MT787040) was composed of 1019 nt, subdivided in a 5’ UTR of 115 nt, a 3’ UTR of 616 nt containing a single instability motif and a polyadenylation signal 21 nt upstream the poly A tail and an ORF of 288 nt. The deduced protein sequence was composed of 95 amino acid residues with a theoretical molecular weight of 10.43 kDa and an isoelectric point of 8.35 (Figure 1B).

The multiple amino acid sequence alignment comparing pmIL-8 with other vertebrates showed that this protein is well conserved among vertebrates, with similarities varying from 85.3% with red-bellied piranha to 37.2% with human IL-8. The putative pmIL-8 protein showed all typical CXC chemokine features, including a 20-residue signal peptide at the N-terminus region and four conserved cysteine residues at positions 32, 34, 58 and 74, with an arginine residue located between the first two cysteines, constituting the typical CXC chemokine structure (Baggiolini *et al.*, 1995) (Figure 2B).

Two predicted disulfide bonds were identified connecting the sulfur atoms between Cys^32^-Cys^58^ and Cys^34^-Cys^74^. As observed for many other teleost IL-8, the ELR motif was not present in the pmIL-8, which showed a Glu-Pro-Arg (EPR) motif in this position instead. The presence of this EPR motif was also observed in the predicted IL-8 sequences of the other characiform species included in this analysis, red-bellied piranha and cave fish (Figure 2B).

The pmIL-8 genomic region (GenBank accession number MT787042) was composed of 2210 nt structurally divided following the same pattern observed for all other vertebrates analyzed, with four exons and three introns. As expected for the untranslated regions, the 5’ and 3’ UTRs showed a higher degree of variation in length, which was also observed for the introns, that varied considerably among species (Figure 3B).

As observed for IL-1β, the pacu IL-8 phylogenetic analysis showed that this protein has a closer relationship with the sequences of the other characiforms, red-bellied-piranha and cave fish. A clear separation between the clades composed by teleost fish and the ones containing other vertebrates, with a further separation based on the orders of the fish was also observed for this gene (Figure 4B).

### Homology modeling of pacu IL-1β and IL-8

For the modeling of the putative pacu IL-1β and IL-8, the amino acid sequences were analyzed using the SWISS-MODEL workspace (Arnold *et al.*, 2006) to identify suitable templates. The search identified 111 significant templates for IL-1β and 326 for IL-8 that matched the target sequences. The crystal structure of human IL-1β and IL-8, that shared 30.20% and 36.76% sequence identity with the pacu sequences, respectively, were selected as templates. The QMEAN global quality scores for the models were −2.29 and −0.67 for IL-1β and IL-8 respectively, indicating that the models had good structural quality.

**Figure 5:**
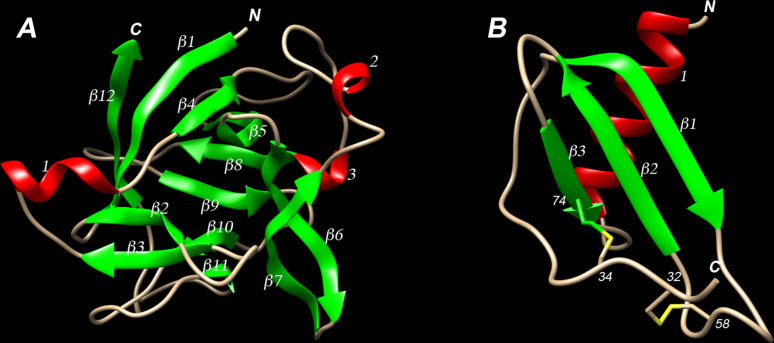
Homology based three-dimensional modeling of pmIL-1β (A) and pmIL-8 (B) presented as ribbon diagrams. Amino- and carboxyl-termini regions are indicated with the letters “N” and “C”, respectively. (A) predicted tertiaty structure of pmIL-1β monomer showing 12 conserved β-strands (green) and 3 α-helices (red). (B) predicted terciary structure of pmIL-8 monomer showing 3 β-strands (green), 1 α-helix (red) and 2 putative disulfide bonds (yellow) between Cys^32^-Cys^58^ and Cys^34^-Cys^74^.

Pacu IL-1β model showed a typical β-trefoil conformation resembling a barrel composed by twelve anti-parallel β-strands and three α-helices with a shallow open face on one end and a closed face on the other, with a central hydrophobic cavity, which is the typical conformational structure of members of the IL-1 superfamily (Nicola, 1994) (Figure 5A).

The predicted pmIL-8 monomeric 3D structure was composed by three anti-parallel β-strands and a single α-helix at the C-terminus region, which is suggested to be involved in the formation of the IL-8 receptor binding site, and is consistent with the known IL-8 structure of other teleosts and human (Clore *et al.*, 1990; Wang *et al.*, 2013; Herath *et al.*, 2016). Another particular common feature of the tertiary structure of pmIL-8 that is shared among many other vertebrates is the occurrence of two putative disulfide bonds between Cys^32^-Cys^58^ and Cys^34^-Cys^74^, which are responsible to stabilize the protein fold (Berkamp *et al.*, 2017) (Figure 5B).

### Tissue distribution of pacu IL-1β and IL-8 transcripts

**Figure 6:**
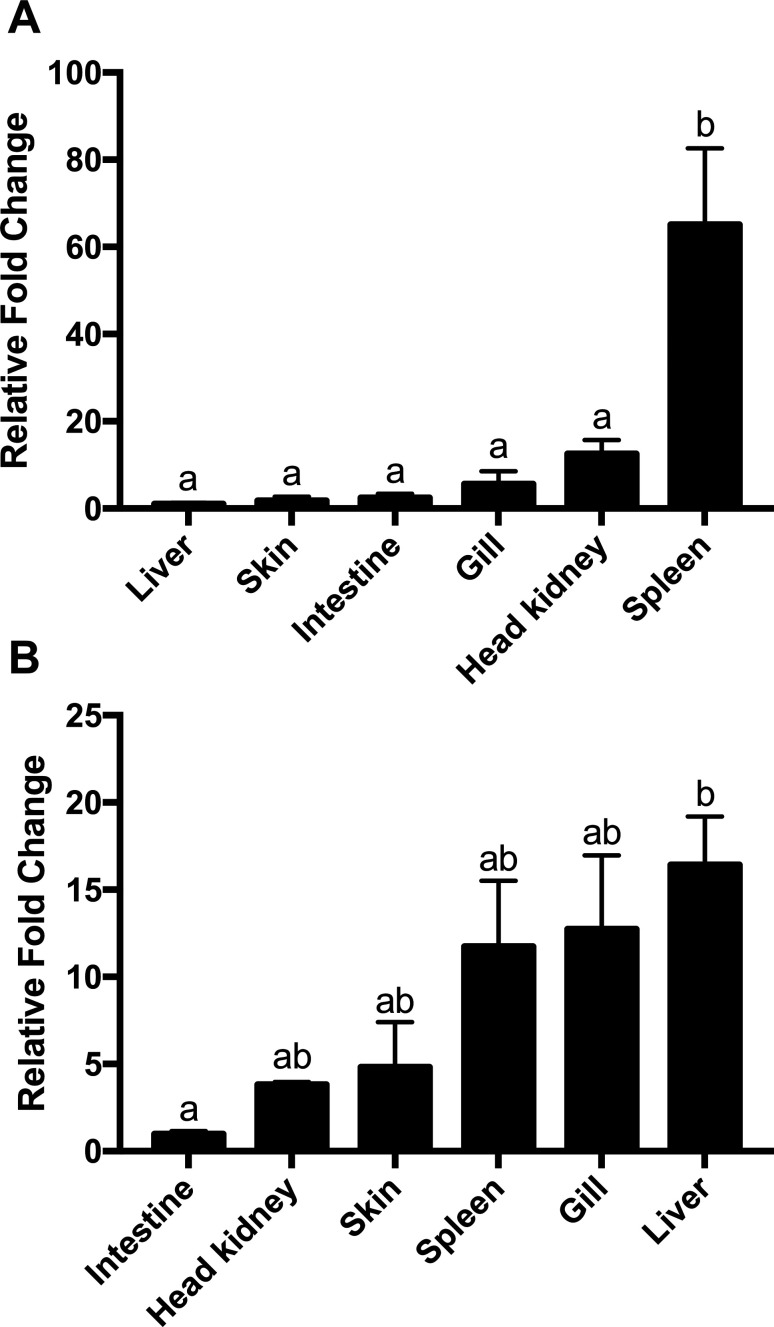
Tissue distribution of mRNA expression of pmIL-1β (A) and pmIL-8 (B) in the liver, spleen, head kidney, gill, intestine and skin of healthy uninfected pacus. Relative mRNA expression is expressed as fold change relative to liver for IL-1β and to intestine for IL-8 calculated using β-actin as endogenous reference. Data are presented as mean ? SEM (n = 5). Levels not connected by same latter are significantly different in Tukey's test (*p* ≤ 0.05).

The tissue distribution of pmIL-1β and pmIL-8 under physiological conditions was investigated by qPCR in the liver, spleen, head kidney, gill, intestine and skin of healthy pacus. Both genes showed constitutive expression in all tissues analyzed with variable amounts of mRNA transcripts. PmIL-1β showed the highest expression level in the spleen, which was 65.1-fold higher than what was observed in the liver, which was the organ with the lowest IL-1β mRNA levels. The other tissues showed consistent lower levels of IL-1β transcripts when compared to the spleen and liver (Figure 6A). For IL-8, constitutive expression was also detected in all tissues analyzed, with the highest expression levels observed in the liver, followed by gill, spleen, skin, head kidney, and with intestine being the organ where this gene was less transcribed (Figure 6B).

### Transcriptional response of *P. mesopotamicus* IL-1β and IL-8 following bacterial infection

To investigate the involvement of the cytokines IL-β and IL-8 under pathogenic conditions in pacu, fish were experimentally infected with a pathogenic strain of *A. dhakensis* and transcripts were quantified in the liver, spleen and head kidney at 12, 24 and 48 h post-infection by qPCR.

**Figure 7:**
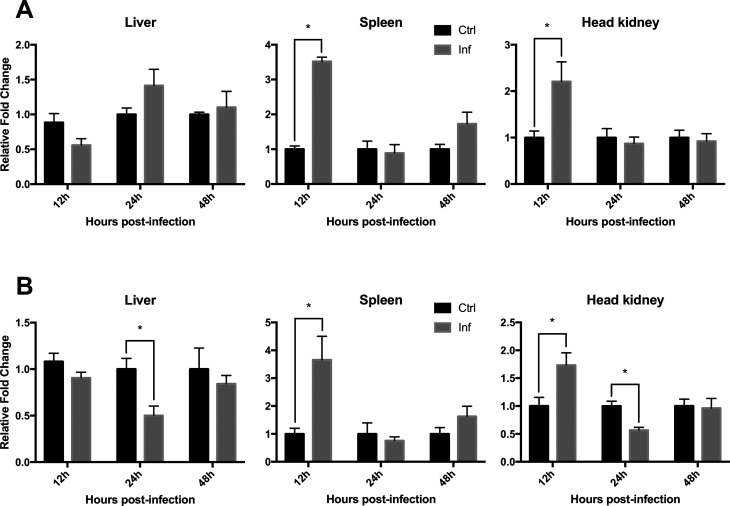
Transcriptional expression levels of pmIL-1β (A) and pmIL-8 (B) in the liver, spleen and head kidney at 12, 24 and 48 h post-infection with *A. dhakensis*. Relative mRNA expression is expressed as fold change to the control samples at the same time point and was calculated using β-actin as endogenous reference. Data are presented as mean ? SEM (n = 5). Asterisks indicate significant differences between control (Ctrl) and infected (Inf) groups in Student's *t*-test (*p* ≤ 0.05).

PmIL-1β showed no significant expression variations in the liver at any of the analyzed periods. On the other hand, significant up-regulation was observed at 12 h post-infection in the spleen (*p* < 0.0001) and head kidney (*p* = 0.0441), where a 3.5 and 2.2-fold transcript increase, respectively, was observed when compared to the control groups. After 24 h, transcript levels did not differ in comparison to the control groups in any of the organs analyzed (Figure 7A).

As observed for pmIL-1β, pmIL-8 showed a significant up-regulation in the spleen (*p* = 0.0331) and in the head kidney (*p* = 0.0319) at 12 h post-infection, again with the first showing higher regulations after the bacterial challenge with 3.65 and 1.73-fold transcript increase, respectively. At 24 h post-infection, on the other hand, this gene showed significant down-regulation in the liver (*p* = 0.0159) and in the head kidney (*p* = 0.0043) to nearly half the expression of the control groups. No expression changes were observed at 48 h post-infection for any of the genes in any of the three organs (Figure 7B).

## Discussion

In the present study, cDNA and genomic DNA sequences of the pro-inflammatory cytokines IL-1β and IL-8 from pacu were characterized and their amino acid sequence, as well as their tertiary structure, were deduced. Furthermore, the transcriptional profile of these proteins was investigated under physiological and pathological conditions.

The genomic structure analysis of pmIL-1β revealed that, even though pacu and human only shared 25.9% identity between their IL-1β amino acid sequences, the exon/intron organization was the same in both species. On the other hand, other fish species with higher amino acid similarity to pmIL-1β, such as zebrafish and rainbow trout, showed different exon/intron organization, with a 6/5 exon/intron structure. This can be explained by the fact that exon/intron structure variations are usually caused by exon fusion, which usually occurs mainly at the 5’ end of the gene, away from the region encoding the biologically active mature peptide (Secombes *et al.*, 2011).

The deduced pmIL-1β precursor peptide was similar in length to the sequences of several vertebrates, with roughly 270 amino acid residues in length that contained the interleukin-1 signature motif is in accordance to the proposed consensus signature for vertebrates [FCL]-x-S- [ASLV]-x(2)-[PSK]-x(2)-[FYLIV]-[LIV]-[SCAT]-T-x(7)-[LIVMK] (Lee *et al.*, 2006). A putative caspase-1 cleavage site was also detected at Asp^114^ producing a putative mature peptide with a molecular weight of 17.21 kDa. This molecular weight is very similar to what is typically observed in mammals, in which IL-1β is known to be expressed as an inactive precursor (proIL-1β) with a molecular weight of about 31 kDa and then cleaved by the enzyme caspase-1 immediately after a conserved aspartic acid located between positions 113 and 117 to form the mature biologically active form with a molecular weight of approximately 17 kDa (Zou *et al.*, 1999; Zhang *et al.*, 2017).

In most teleost species, the cleavage mechanism involved in the processing of pro-IL-1β into the mature peptide is not fully elucidated, since a caspase-1 cleavage site identical to what is observed in mammalian IL-1β has not been identified in any fish species so far (Hong *et al.*, 2004; Pelegrin *et al.*, 2004). However, there are some fish species, such as sea bass, in which IL-1β precursor has been shown to be specifically cleaved by caspase-1 in a different aspartic acid residue than mammalian IL-1β to produce the 18 kDa biologically active mature peptide by cleavage of its pro-form at Asp^100^ (Reis *et al.*, 2012). When comparing the IL-1β sequences of pacu and sea bass, a conserved aspartic acid (Asp^123^) was observed in the same position as the putative cleavage position of sea bass, which would suggest that this is in fact the cleavage site.

However, in the homology modeling analysis using human IL-1β as template, this position was located in a beta sheet-forming region, which has important structural function in mammalian and chicken IL-1β. For this reason, although further functional experiments are still needed to confirm the actual caspase-1 cleavage site in pmIL-1β, Asp^114^ was considered more likely to undergo cleavage by caspase-1 than Asp^123^. It is important to point out that sea bass IL-1β does not have a homologous aspartate in the position of pmAsp^114^, which has led to the deduction that, for this species, Asp^100^ is the caspase-1 cleavage site (Reis *et al.*, 2012).

Moreover, the pmIL-1β showed a typical β-trefoil structure consisting of twelve antiparallel β-strands and three α-helices, forming a barrel-like structure with a central hydrophobic cavity, which is the typical conformational structure of members of the IL-1 superfamily. Even though this hydrophobic cavity is not directly implied to any functional aspect of its pro-inflammatory properties, it though to probably play a critical role in maintaining the structural integrity of the protein (Cheng *et al.*, 2011). It is important to point out that, this typical structure only is observed when considering Asp^114^ as the caspase-1 cleavage site, similar to what is observed in mammals, and not Asp^123^, which is in the same position as the cleavage site observed in sea bass IL-1β. In this case, only eleven β-sheets would be observed and consequent destabilization of the other C-terminal beta-sheet forming segments could occur (Reis *et al.*, 2012).

The genomic DNA sequence of pmIL-8, composed of four exons and three introns, was the same observed for all fish species analyzed in the present study, as well as for humans. The exon lengths, as well as the overall amino acid sequence structure of pmIL-8, were also highly similar to other vertebrates, indicating that this chemokine is evolutionarily conserved across vertebrates (Berkamp *et al.*, 2017).

The deduced amino acid sequence of pacu IL-8 showed a signal peptide and four conserved cysteine residues, which are consistent with the typical CXC chemokine structure and containing, as observed for most species, less than one hundred amino acid residues (Baggiolini *et al.*, 1995; Berkamp *et al.*, 2017). The amino acid arginine observed between the first two cysteine residues is also conserved in almost all teleosts with few exceptions, such as the large yellow croaker (*Larimichthys crocea*), which has a glutamine in this position (Wang *et al.*, 2013).

In mammalian IL-8, the ELR motif is usually observed right before the CXC motif and is recognized as being involved in the neutrophil attraction properties of this chemokine (Sabat and Schwartz, 2004). In general, this motif is vastly diverse among IL-8 from teleosts, with some species containing the same ELR motif found in mammals, such as haddock and Atlantic cod, while others have a SLH, PLR, DLR or LLR motif in this position (Lee *et al.*, 2001; Fujiki *et al.*, 2003; Corripio-Miyar *et al.*, 2007; Harun *et al.*, 2008; Seppola *et al.*, 2008; Oehlers *et al.*, 2010; Li and Yao, 2013; Chu *et al.*, 2014). PmIL-8, however, did not show any of the aforementioned motifs, showing a unique EPR motif in this position, which was shared only by the species red-bellied piranha and cave fish, also members of the Characiformes order, suggesting that this motif is conserved only among representatives of this particular order.

Even though teleost IL-8 usually do not have a classical ELR motif, it has already been shown that this cytokine still exerts a chemoattractant effect on neutrophils and mononuclear leukocytes, and induces neutrophil infiltration, which indicates that this motif is not strictly necessary to produce its chemoattractant and pro-inflammatory activities in fish (Sun *et al.*, 2011; van der Aa *et al.*, 2012; Wang *et al.*, 2013). This was observed in the present study, where a significant upregulation of this protein in bacteria-infected fish was observed (further discussed below), indicating that, even without the presence of the ELR motif, pmIL-8 is still highly involved in the initiation of the inflammatory process. Nevertheless, the exact influence of each of the motifs in the chemoattractant effect in teleost IL-8 still needs further investigations.

The molecular structure of pmIL-8 was composed of three antiparallel β-strands as well as a single α-helix, which is suggested to be involved in the formation of the IL-8 receptor binding site, and is consistent with the known IL-8 structure of other teleosts and humans (Clore *et al.*, 1990; Wang *et al.*, 2013; Herath *et al.*, 2016). Another particular common feature of the tertiary structure of pacu IL-8 that is shared among many other vertebrates is the occurrence of two disulfide bonds linking the conserved cysteines that stabilize the protein fold (Berkamp *et al.*, 2017). The observed structural similarity of pmIL-8 with other previously described IL-8 corroborates the idea that this protein shares similar biological function with other vertebrate counterparts.

The tissue distribution of pmIL-1β and pmIL-8 transcripts in the liver, spleen, head kidney, gills, intestine and skin of healthy pacus showed that both cytokines are constitutively expressed in all six tissues examined, with higher levels of IL-1β being observed in the spleen, followed by the head kidney, and with similar expression level among all other tissues, whereas the highest transcript amounts of IL-8 was observed in the liver, followed closely by gill, spleen, skin and head kidney.

Such pleotropic distribution of IL-1β and IL-8 across several tissues of healthy fish has already been described for other teleost species (Pelegrin *et al.*, 2001; Scapigliati *et al.*, 2001; Engelsma *et al.*, 2003; Chen *et al.*, 2005; Qiu *et al.*, 2009; Wang *et al.*, 2013), suggesting that these cytokines are probably involved in biological processes other than the response to pathogenic infections. Furthermore, the distribution pattern of these genes is not consistent in all fish species, which indicates that interleukin mRNA expression profile is species-specific (Herath *et al.*, 2016).

In the analysis investigating the transcriptional effects of the bacterial infection with *A. dhakensis*, it was clearly observed that, for both genes, the spleen was the organ that showed higher up-regulation in response to the bacterial infection, followed by the head kidney. This could be explained by a higher leukocyte production in the spleen, or it could only reflect the natural progression of the infection throughout the organs. A similar transcriptional behavior was observed for IL-1β and IL-8 under bacterial- and/or LPS-stimulation in channel catfish (Chen *et al.*, 2005; Wang *et al.*, 2006), sea bream (Pelegrin *et al.*, 2004), black rockfish (Herath *et al.*, 2016) and Japanese sea perch (Qiu *et al.*, 2009), in which high transcript upregulation was observed in the spleen and head kidney. These results were to be expected, given that this cytokine is highly produced by leukocytes in the early phases of the inflammatory process (Dinarello, 2010), which in the case of *Aeromonas* infections in *P. mesopotamicus*, takes place at around 12 h post-infection.

Similar to what was observed in the present study, where a significant decrease in pmIL-8 transcript levels was observed in the liver and head kidney, a significant downregulation of IL-8 was observed in the spleen and blood cells of black rockfish stimulated with *Streptococcus iniae* and poly I:C after 24 h post-stimulation and following an initial upregulation (Herath *et al.*, 2016). This is probably due to the activation of anti-inflammatory pathways of monocytes and macrophages which are activated to prevent tissue damage during acute inflammation, mainly by the production of anti-inflammatory cytokines, such as interleukin-10, which is known to induce down-regulation of pro-inflammatory cytokines like IL-8 (Sabat *et al.*, 2010).

Also indicated by the findings of the present study and for many other teleost species, the expression profiles of IL-8 under inflammation indicates that the presence of the ELR motif apparently is not strictly necessary for it to exert chemotactic activity in the inflammatory responses against pathogens in teleosts. On the other hand, the other important function related to the presence of the ELR motif, which is the formation of new blood vessels known as angiogenesis, is not fully explored in fish. (Strieter *et al.*, 1995) have shown that, in mammals, CXC chemokines that contain the ELR motif showed potent angiogenic activity, whereas CXC chemokines lacking this motif where highly angiostatic. Therefore, further studies are needed to evaluate the angiogenic/angiostatic properties of the EPR motif observed in pacu IL-8 in comparison to the classical ELR motif found in mammals.

Taken together, the results obtained in the present study suggest that IL-1β and IL-8 are involved in the early response against bacterial pathogens in pacu, which, during bacterial infections, takes less then 12 hours to initiate, and since the inflammatory process occurs as a cytokine activation cascade, after this period, their expression decreases to physiological levels and other proteins may take place in the maintenance of the inflammatory process (Secombes *et al.*, 2011).

In summary, the molecular cloning and characterization of the genomic DNA and full-length cDNA of the cytokines IL-1β and IL-8 from *P. mesopotamicus* are provided in the present study showing that these cytokines have structural similarities with other teleost species, but also present some features that appear to be specific for fish from the Characiformes order. Furthermore, transcript analysis indicates that these interleukins are constitutively expressed in all organs analyzed, with the highest levels found in the spleen for IL-1β and in the liver, gill and spleen for IL-8. Under pathogenic conditions, these genes are involved in the early response against bacterial infections, mainly in the spleen and head kidney.

To the best of our knowledge, this is the first comprehensive study investigating the biological properties of these two important cytokines in a fish from the Characiformes order, further contributing to a better understanding of the mechanisms coordinating the initial response against bacterial pathogens in teleosts.
